# Implementation of risk-reducing surgery for HBOC under public insurance in Japan: a single-center experience

**DOI:** 10.1007/s10689-025-00501-8

**Published:** 2025-10-16

**Authors:** Hiroko Terui-Kohbata, Sayako Takahashi, Eriko Takamine, Mariko Komine, Maki Gau, Makiko Egawa, Yusuke Ebana, Tomoyuki Aruga, Kimio Wakana, Masayuki Yoshida

**Affiliations:** 1 Department of Medical Genetics, Institute of Science Tokyo Hospital, Tokyo, Japan; 2 Department of Life Science and Bioethics, Graduate School of Medicine, Institute of Science Tokyo, 1-5-45 Yushima, Bunkyo, Tokyo, 113-8510 Japan; 3 Department of Nutrition and Metabolism in Cardiovascular Disease, Graduate School of Medical and Dental Sciences, Institute of Science Tokyo, Tokyo, Japan; 4 Department of Breast Surgery, Institute of Science Tokyo Hospital, Tokyo, Japan; 5 Perinatal and Gynecological Department, Institute of Science Tokyo Hospital, Tokyo, Japan

To the Editor,

In Japan, *BRCA1/2* genetic testing was publicly insured in 2018, followed by risk-reducing salpingo-oophorectomy (RRSO) in 2020 and risk-reducing mastectomy (RRM) with reconstruction in 2022. These policy changes are considered to have expanded access to hereditary cancer prevention, although uptake and timing may still be influenced by clinical and personal factors. Therefore, we investigated the implementation of preventive surgery among women with hereditary breast and ovarian cancer syndrome (HBOC) at our institution under public insurance coverage.

At our institution, 23 women with HBOC underwent preventive surgery under public insurance between April 2020 and December 2024. Among them, 15 (65.2%) underwent RRM, 18 (78.3%) underwent RRSO, and 10 (43.5%) received both. Of these 23 women, 19 had been diagnosed with HBOC at our genetics department, and 10 of these 19 (52.6%) proceeded to preventive surgery.

The mean ages at RRM and RRSO were in the mid-40 s, with no significant differences between *BRCA1* and *BRCA2* carriers. The average interval from diagnosis to surgery was 6.5 months for RRM and 11.3 months for RRSO. Importantly, RRSO was significantly delayed among *BRCA2* carriers compared with *BRCA1* carriers (15.2 *vs.* 6.4 months, *p* = 0.02). The only three women who have continued to defer RRSO did so due to fertility considerations.

Before insurance coverage, only one or two RRSO procedures were performed annually, and RRM was rare because costs exceeded JPY 1,000,000, including reconstruction. Public insurance thus played a pivotal role in expanding access to both procedures [1, 2]. The observed delays among *BRCA2* carriers likely reflect later ovarian cancer onset and fertility considerations [3]. These findings highlight the importance of individualized counseling that considers age, reproductive planning, and gene-specific risk, and multicenter studies are warranted to confirm whether our single-center experience reflects national trends.

## Data Availability

No datasets were generated or analysed during the current study.
